# Effectiveness of a structured motivational intervention including smoking cessation advice and spirometry information in the primary care setting: the ESPITAP study

**DOI:** 10.1186/1471-2458-11-859

**Published:** 2011-11-11

**Authors:** Francisco Martin-Lujan, Josep Ll Piñol-Moreso, Nuria Martin-Vergara, Josep Basora-Gallisa, Irene Pascual-Palacios, Ramon Sagarra-Alamo, Estefania Aparicio Llopis, Maria T Basora-Gallisa, Roser Pedret-Llaberia

**Affiliations:** 1 Study Group on Respiratory Tract Diseases (GEPAR), Primary Care Research Institute (IDIAP) Jordi Gol, Barcelona, Spain; 2 School of Medicine and Health Sciences, Rovira Virgili University, Tarragona, Spain; 3 C/Camí de Riudoms, 53-55. Reus-43203, Tarragona, Spain

## Abstract

**Background:**

There is current controversy about the efficacy of smoking cessation interventions that are based on information obtained by spirometry. The objective of this study is to evaluate the effectiveness in the primary care setting of structured motivational intervention to achieve smoking cessation, compared with usual clinical practice.

**Methods:**

**Design**

Multicentre randomized clinical trial with an intervention and a control group.

**Setting**

12 primary care centres in the province of Tarragona (Spain).

**Subjects of study**

600 current smokers aged between 35 and 70 years with a cumulative habit of more than 10 packs of cigarettes per year, attended in primary care for any reason and who did not meet any of the exclusion criteria for the study, randomly assigned to structured intervention or standard clinical attention.

**Intervention**

Usual advice to quit smoking by a general practitioner as well as a 20-minute personalized visit to provide detailed information about spirometry results, during which FEV1, FVC, FEF 25-75% and PEF measurements were discussed and interpreted in terms of theoretical values. Additional information included the lung age index (defined as the average age of a non-smoker with the same FEV1 as the study participant), comparing this with the chronological age to illustrate the pulmonary deterioration that results from smoking.

**Measurements**

Spirometry during the initial visit. Structured interview questionnaire administered at the primary care centre at the initial visit and at 12-month follow-up. Telephone follow-up interview at 6 months. At 12-month follow-up, expired CO was measured in patients who claimed to have quit smoking.

**Main variables**

Smoking cessation at 12 months.

**Analysis**

Data will be analyzed on the basis of "intention to treat" and the unit of analysis will be the individual smoker.

**Expected results**

Among active smokers treated in primary care we anticipate significantly higher smoking cessation in the intervention group than in the control group.

**Discussion:**

Application of a motivational intervention based on structured information about spirometry results, improved abstinence rates among smokers seen in actual clinical practice conditions in primary care.

**Trial registration:**

ClinicalTrial.gov, number NCT01194596.

## Background

Smoking is the principal avoidable risk factor and etiological cause of numerous pathologies, including respiratory tract [1]. Smoking accelerates the physiological decline of pulmonary volume usually attributable to age, and the most susceptible subjects may develop chronic obstructive pulmonary disease (COPD) [2]. The estimated absolute risk of COPD for smokers is at least 25% [3]. The prevalence of COPD increases with age and is more frequent in men, but this appears to be related to the cumulative effect of other risk factors to which the individual has been exposed during a lifetime [4]. Spirometry is considered the standard test for the diagnosis and monitoring of COPD [5,6]; nevertheless, it appears to be an inefficient screening tool for the general population. Some institutions advise against its systematic use in asymptomatic subjects for various reasons, not only cost-benefit considerations [7]. In addition, the available evidence seems to indicate that the information spirometry provides does not modify COPD management in the early stages of disease [8], when quitting a smoking habit is the most effective therapeutic intervention and the only one that increases survival [1]. Therefore, early intervention is considered crucial.

From a therapeutic perspective, smoking is a chronic disease of an addictive nature, with frequent relapses that reflect the intensity of nicotine dependence. The available data suggest that most smokers are interested in quitting and that structured advice from health professionals with whom they are in contact is an important motivating element [9]. Therefore, interventions based on individual or group counselling should be the first step in smoking cessation, with the addition of pharmacological treatment when educational strategies have not been effective [10]. In fact, the combination of advice and medication is more likely to succeed than either approach in isolation. In patients with COPD, a recent study indicates that rates of abstinence increase as interventions are added, from 2.6% with brief counselling to 12.3% when combined with drug therapy [11]. However, while it is true that adding medication significantly improves abstinence, cessation efforts often fail because of the lack of motivation of smokers [12]. In an effort to improve success rates, spirometry was advised as a motivational tool to reinforce the smoking cessation message provided by counselling [13].

Some years ago, the Lung Health Study showed that COPD could be detected early through the use of spirometry and established the benefits of smoking cessation for changing the natural history of the disease [14]. In addition, the quit rate among participants included in the intensive program was 21.7%, compared to 5.4% among subjects in the standard program [15,16]. Bednarek et al. [17] have also published results obtained in a cohort of 4494 smokers in Poland who received a minimal intervention, placing their spirometry data on the classic Fletcher-Peto graph [18]. After 1 year of follow-up, the cessation rate was significantly higher in patients with COPD than in subjects with normal spirometry (16.3% vs 12.0%; *P *= 0.0003). Therefore, it is logical to assume that information about respiratory function testing could also be helpful. However, these results are from observational studies lacking sufficient statistical power to support any recommendations, leading some authors to find the evidence of the motivational merits of spirometry testing to be less than convincing [19,20].

At present, the use of spirometry as a motivational tool for smoking cessation continues to be a controversial topic. The most recent Cochrane review of the assessment of biomedical risks as a quit-smoking aid insists that, due to the scarcity of high-quality analyses, no definitive conclusions can be reached about the effectiveness of this approach [21]. However, it also points out that spirometry had a significant effect in the only high-quality study, published in 2008 by Parkes et al. [22]. This clinical trial included 561 smokers older than 35 years who, after spirometry testing, were randomized to study groups, one of which received a summary of the results, describing their "lung age" and comparing it with their chronological age. After 12 months follow-up, abstinence rates confirmed by urinary cotinine levels were significantly higher in the intervention group (13.6% vs 6.4%, *P *= 0.005) and these subjects had a higher probability of abandoning their habit than those who did not receive this explanation (RR: 2.12; IC95%: 1.24-3.62). Moreover, Kotz et al. published a clinical trial in 2009 with the hypothesis that early detection of COPD and the provision of information about the results could be more effective than standard efforts to help smokers to quit [23]. The study evaluated 296 smokers aged 35-70 years with COPD detected by spirometry testing, who were randomly assigned to either confrontational counselling for smoking cessation in which a nurse respiratory specialist provided feedback on the results (experimental group) or "care as usual" for smoking cessation delivered by the general practitioner. After 52 weeks of follow-up, the intervention group achieved higher abstinence rates (11.2% vs 5.9%), although without reaching statistical significance (OR: 2.02; IC95%: 0.63-6.46). These studies appear to present contradictory results, although congruence emerges if we analyze them in detail: the use of spirometry in "healthy" smokers increases motivation (and the probability of quitting smoking), while confrontation with their lung function data is less effective in subjects with COPD. More recently, in 2010, McClure et al. [24] published a clinical trial with 536 smokers, recruited from the general North American population, who were randomized to receive a brief motivational intervention for smoking cessation (≈20 min), including spirometric testing and feedback; no differences in abstinence rates were found at 1 year of follow-up. However, in a later publication they reported that the participants with changes in their lung function tests made greater use of quit-smoking services and achieved an abstinence rate almost double that of controls at 6 months (17.2% vs 8.9%, *P *= 0.05; OR: 2.13, IC95%: 1.04-4.5), although the effect was diluted at 12 months (16.2 vs 11.3, *P *= 0.26; OR: 1.52, IC95%: 0.73-3.1) [25]. Therefore, given these other results, they concluded that more research is NEEDED to determine whether the impact of spirometry feedback differs according to the deterioration in the smoker's pulmonary function.

## Objectives

The main objective of this study is to evaluate the effectiveness in the primary care setting of a structured motivational intervention and feedback on spirometry data to achieve smoking cessation, compared with usual clinical practice and assessed with respect to quit rates at 12 months after the intervention.

## Methods

### Design

**Figure 1:**
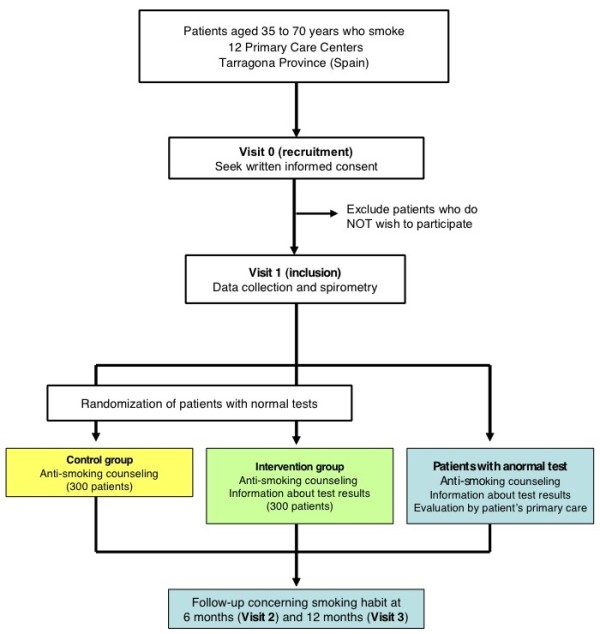
**Diagram of the ESPITAP study: process of selection, randomization and follow-up of subjects included in the study**.

A randomized, controlled clinical trial is proposed, enrolling current smokers with no previous history of respiratory disease in the Primary Care Centres of the province of Tarragona (Spain). The general design of the study is detailed in Figure 1. This trial has been registered with ClinicalTrial.gov (NCT01194596).

### Setting and study sample

The study population will be recruited during consultations in 12 primary care practices in the province of Tarragona (Spain) managed by the Catalan Health Institute, 8 of which are urban and 4 rural, serving an adult population (aged > 18 years) of 195, 343 patients. Candidates for participation are smokers aged 35 to 70 years who visit a centre for any reason during regular office hours, and who meet all inclusion criteria and none of the exclusion criteria.

- *Inclusion criteria*: current smoker, with a cumulative consumption of more than 10 packs/year.

Current smoker is defined as having smoked daily during the past month, regardless of the quantity. Cumulative consumption is defined as the daily average of cigarettes smoked, multiplied by the number of years of smoking, divided by 20.

- *Exclusion criteria*:

1) Any evidence of previous diagnosis of a respiratory disease,

2) Functional pulmonary testing conducted within the previous 12 months,

3) Presence of any chronic or terminal condition that changes the baseline parameters or complicates the testing and analysis to be conducted during the study period,

4) Impossibility of completing follow-up for any reason,

5) Patient refusal to continue with the study.

### Measurements

In the recruitment visit (visit 0), all potential subjects will be advised that smokers are susceptible to COPD and that spirometry is a simple, non-invasive test that permits the detection of this disease at an early stage. They will then be informed about the study and offered the possibility of participating. Those who accept will be asked to provide their signed informed consent. Consenting participants will then be asked for all of the necessary information using a structured data collection questionnaire designed for this purpose, which will include:

• Affiliations and sociodemographic data.

• History of diseases and medications.

• History of respiratory symptoms.

• Smoking habit: current daily consumption (cigarettes/day) and accumulated consumption (packs/year averaged over years of smoking), nicotine dependence measured by the Fagerström test [26], motivation to quit smoking using the Richmond test [27] and stage of the change process according to Prochaska and DiClemente's model [28].

• Previous quit attempts: number and therapeutic resources used.

• Basic physical examination: weight, height, body mass index, blood pressure.

• Expired carbon monoxide (CO) levels determined by coximetry using the Smoke Check device (Micro Medical Limited, Rochester, Kent, England). This model detects a CO range of 0-20 particles per million (ppm) with a sensitivity of 1 ppm.

• Spirometry and a bronchodilator test, using an ultrasound pneumotacograph Datospir-600 (SIBELMED, S.A.).

### Allocation of study groups

All patients will be scheduled for assessment of their lung function, using spirometry, at their primary care centre. The quality of testing will be ensured by a three-tiered system of quality control:

1) Standardization of pulmonary function testing. Only tests that meet the quality recommendations and criteria of the *American Thoracic Society-European Respiratory Society *[29,30] will be accepted as valid:

a) At least three attempts will be made, with the selection of the best values of forced vital capacity (FVC), forced expiratory volume in 1 s (FEV1), and FEV1/FVC ratio;

b) Variability between these measures must be less than 5%, exhalation time greater than 6 s, and the attempt must be properly initiated, with a rapid increase;

c) Airflow-limitation reversibility will be determined and considered positive if it is greater than 12% and improvement in FEV1 > 200 ml, observed after the administration of 400 micrograms of salbutamol inhaled with a valve-holding chamber (spacer).

2) Technical considerations. To facilitate access, all testing will be conducted at each participating centre by technicians selected from the nursing staff, who must be able to show specifically accredited training from the Health Research Institute of Catalonia.

3) Centralized supervision. All testing will be sent to one reference observer who will be responsible for centralized quality control and will interpret the results by following an automated process using a previously validated and registered computer program (ESPIRO) [31]. Finally, a clinical report will be generated and analyses will be considered normal if they meet the following criteria:

- FEV1 ≥ 80% of the theoretical reference value

- FVC ≥ 80% of the theoretical reference value

- FEV1/FVC ≥ 0.7

Subjects with test results within normal range will be allocated to receive or not the proposed intervention. The assignment sequence will be centralized at the Research Support Unit of the IDIAP Jordi Gol in Tarragona and will be blinded and performed consecutively, following a simple, numerical relationship randomization issued for this purpose. Due to the nature of the intervention it is impossible for the patient or the researcher to be blinded to the assignment.

#### Description of the intervention and control group

Within one month of their random assignment, all participants will be contacted to schedule their intervention visit (visit 1), during which they will receive health education and counselling on smoking cessation from their physician (Table 1). Control group subjects will have a brief visit (5-10 min) in the format that is usually recommended for primary care professionals [32]: a clear, firm, personalized proposition recommending that they quit smoking, in an empathic and respectful context.

**Table 1:** Smoking cessation counseling protocol

Smoking cessation counseling protocol
Control group counseling: brief intervention

*"As your doctor, I recommend that you quit smoking, although the decision is yours alone. I know that it is difficult to make a decision right now, but we have a number of resources that can help you quit smoking and I know that if you decide to quit in the future you will be able to do that. Remember that you can always count on us to help."*

Intervention group counseling: detailed discussion of spirometry

*"Tobacco is harmful to your health. Smoking can produce various diseases such as, for example, those that affect your lungs. Some smokers end up getting a chronic pulmonary disease that is commonly called chronic bronchitis. To diagnose that disease, we use a test called spirometry that measures lung capacity. This is the test I gave you. In your case, the results indicate that you do not have this disease now, but some of the data suggest that tobacco is causing some changes in your lungs*.

*These values - FVC, FEV_1_, FEV_25-75 _- indicate that you have lost part of the capacity you had to exhale air, specifically, you have lost about..........%*.

*Tobacco also accelerates the usual aging of the lungs. This effect can be seen in this "lung age" value, which is....... years*.

*In summary, although at this time, given your results, we would not say you are sick, looking at the rest of the data, the best thing for your health is to stop smoking as soon as possible."*

Patients assigned to the intervention group will receive a written spirometry report and a detailed, structured explanation of the content during a personalized visit of 15 to 20 min, using a motivational interview approach. Researchers at the participating centres will come to a consensus on the comments about spirometry to be delivered, and these will focus on a standardized description of FEV1, FVC, forced expiratory flow between 25% and 75% of the FVC (FEF25-75%) and peak expiratory flow (PEF) values and their interpretation in terms of their theoretical value. In addition, they will inform subjects about the lung age index (defined as the average age of a non-smoker with the same FEV1 value as the subject in question) [33], comparing this value with the chronological individual age to illustrate the lung damage suffered as a consequence of smoking. In practice, the lung age is automatically generated by configuring the spirometer. The Fletcher-Peto graph [18] will be used to depict how the loss of lung function that occurs with age can be accelerated in smokers and how quitting smoking normalizes the pace of pulmonary deterioration, even though the lost capacity cannot be recovered.

With respect to patients with changes in their test values (FEV1 and/or FVC < 80% of the theoretical value and/or FEV1/FVC < 0.7), all will be informed about them and also referred to their physician for evaluation, verification of test results, and clinical follow-up according to the standard practice at each centre.

Independent of their group assignment, all participants who show an interest in quitting smoking will be offered a treatment option in the smoking cessation program that forms part of the usual activities of each centre.

Both physicians and nurses at the participating centres will receive support and at least 8 h of specific training on spirometry and motivational interview techniques, applied to smoking addiction, which will be accredited by the Catalan Health Institute.

#### Follow-up period

All subjects will be evaluated during a telephone follow-up at 6 months post-intervention (visit 2) to determine whether there has been any change in their smoking status and to record new data about tobacco consumption or, where applicable, to record quit date. Finally, at 12 months after inclusion in the study, they will be scheduled for their final study visit (visit 3), which will take place at the corresponding centre. At this last visit, new data will be collected on each subject's smoking habit (current consumption, dependence, motivation and stage in the change process) or, where applicable, quit date. Patients who claim to have quit smoking will undergo a breath test to measure expired CO.

#### Main outcome variables

The primary variable will be prolonged abstinence from smoking, and a secondary variable occasional abstinence (point prevalent abstinence), both of which will be validated using confirmation by coximetry at 12 months post-intervention. The cut-off point to confirm abstinence will be 10 ppm, since lower values indicate a non-smoker and higher values reflect having smoked within the preceding 12 to 24 h [34].

The criterion to establish abstinence takes into account the Society for Research on Nicotine and Tobacco recommendations and definitions [35]:

- *Continuous abstinence *refers to sustained abstinence between the point of intervention and a follow-up point.

- *Prolonged abstinence *refers to sustained abstinence from an initial "grace period" until a follow-up point. The "grace period" is understood as the time immediately after a definite quit date or intervention during which continued smoking is not considered failure to quit. For most studies, the recommendation is that this period not exceed 2 to 4 weeks.

- *Point prevalence abstinence *refers to abstinence during a time window immediately before the follow-up point (usually 7 days).

- *Failure to quit *refers to a smoking relapse (of any type) lasting 7 consecutive days (defined by the U.S. National Heart, Lung, Blood Institute).

In general, continuous abstinence is accepted as the *gold standard*, but this is a very rigorous measure; therefore, prolonged abstinence is preferred because it allows inclusion in the analysis of subjects who achieve a sustained abstinence after a transitional "grace period" and whose effort would otherwise be considered failure.

#### Sample size calculation

To achieve the study's main objective, sample size is calculated in accordance with the following parameters: identification of a difference equal to or greater than 8% between the intervention group and control group with regard to abandonment or reduction of tobacco use, a 10% abandonment or reduction in the control group, an alpha risk of 0.05, and a beta risk of 0.20 in a two-tailed contrast. Consequently, for this study it will be necessary to randomize a total of 590 subjects (295 in each group). Taking into account a withdrawal rate of 10%, the total sample size will be 649 subjects.

At each of the 12 participating centres, it should be reasonable to expect the associated researchers to include about 50 participants in this study (25 in each group).

#### Analysis strategy

Data will be drawn from a centralized database and grouped so that those responsible for analysis will be blinded to participants' study group assignment.

The effectiveness of randomization to assess the comparability and consistency of intervention and control groups will be checked in terms of the similarity of the distribution of the variables of interest at baseline. Losses to follow-up in each group will be quantified and assessed to rule out a possible selection bias.

All analysis will be conducted on the principle of intention to treat. Losses to follow-up will be quantified in each group and assessed to determine whether any possible bias was introduced to the results. A "worst-case analysis" strategy will be used, assuming that the expected response occurred in the control group patients lost to follow-up and did not occur in the intervention group. Using this assumption, the data will be reanalyzed and the magnitude of the observed difference between the initial results and the "worst-case" estimates will be assessed. This approach is valid except in the unlikely case that losses to follow-up exceed 20%.

Standard statistical tests will be used for both quantitative and qualitative variables described (e.g. means, standard deviations, medians, proportions).

Continuous abstinence, prolonged abstinence rates, and point prevalence abstinence at 6 and 12 months after intervention will be calculated, along with Cox analysis of survival (abstinence). In cases of failure to quit, consumption data, motivation, and number of attempts to quit smoking during follow-up will be collected and compared with those who achieved a level of abstinence.

The results will be presented using both relative and absolute measures: relative risk (RR) and relative risk reduction (RRR) and absolute risk reduction (ARR) and the number needed to treat (NNT), or number of smokers who must try to quit to succeed in getting one to remain abstinent. All measures will be expressed with their respective confidence intervals (CI) of 95%.

To assess the influence of studied parameters on the rates of abstinence and failure, logistic regression will be used, calculating odds ratios (OR) and 95% CI. As part of the statistical analysis, multiple logistic regression models will be fitted to adjust for potential confounding covariates of the primary outcome (age, sex, educational level, number of previous quit attempts, nicotine dependence, motivation to quit smoking, comorbidity, etc.) [36,37].

Statistical analysis will be performed with SPSS version 14.0. The tests will be considered significant when *P *< 0.05.

#### Ethical aspects

The protocol, informed consent form, participant information sheet and any applicable documents were submitted to an appropriate Ethics Committee (EC) and Regulatory Authority of Institute of Primary Care Research (IDIAP) Jordi Gol for written approval and the study protocol was approved by the registration number ID 4R07/040. All substantial amendments to the original approved documents will also be sent to an appropriate EC and regulatory authority for written approval.

Investigators will ensure that this study is conducted in accordance with the principles of the Declaration of Helsinki and ICH Guidelines for Good Clinical Practice, and in full conformity with relevant regulations.

All participants will be informed of the study, its objectives and activities related to their participation: number and schedule of visits, diagnostic test, results information, etc.

Written information will be given and they will be asked to sign a consent form.

Patients with any previously undiagnosed impairment will be referred to the appropriate diagnostic and therapeutic unit, which will be responsible for integrating information and providing the best diagnostic and therapeutic option.

The trial staff will ensure that participant confidentiality is maintained. Participants will be identified only by a PID number on the CRF and any electronic database. All documents will be stored securely and only accessible by trial staff and authorized personnel. The study will comply with the Data Protection Legislation requirements for anonymization of data.

## Discussion

The etiological relationship between COPD and smoking is no longer subject to discussion due to the available epidemiological, morphological and genetic evidence. There is no room for doubt that quitting smoking is the best way to reduce the risk of COPD, and is the most efficient intervention and the only effective treatment to improve survival [1]. However, smoking requires an intensive approach that is not always viable under the actual conditions of everyday clinical practice, which is often consumed with massive numbers of patient visits. Therefore, it is of fundamental importance to have evidence of ways to maximize these interventions.

The present study will evaluate, under nearly actual conditions of primary care, the efficacy of a quit-smoking strategy directed at individuals at any of the stages of change. The theoretical framework used is to provide personalized motivational information about the effects of smoking, with the goal of accelerating a change in habit. This idea is not new and was the subject of a recent Cochrane review [21]. Although certain prestigious authors and institutions [8,20] have raised questions about the usefulness of this type of strategy, recent data allow us to assume that some patients can benefit from it [22,25]. One plausible explanation of this apparent contradiction is that if an intervention is to succeed there must be some change in testing results and an adequate presentation of them [37]. If this were the case, being able to prove a change should have implications for clinical practice.

Primary care, with its population context and continuity of care throughout a patient's life, has a core role in any strategy to break a smoking habit. One of the main tasks of first-line care professionals is to increase patient motivation to quit smoking, and on many occasions it must be attempted in young, healthy smokers that have come to the doctor for some other reason. The ESPITAP study presents a pragmatic design that reflects these conditions, attempting to establish an intervention that can be easily applied in clinical practice. For that reason, it takes into account the usual attention these subjects receive and is intended to be incorporated into the daily clinical activity of the researchers.

The designed intervention is less intense than the one described in other studies that have taken a motivational approach, and this is not of marginal importance: a direct dose-response association has been demonstrated between intensity, success of the intervention, and abstinence rates [21]. Although one could then assume that another more intensive approach would be more appropriate, at the same time this would distance us from the conditions of daily practice that the study attempts to reflect.

A primary strength of the study is its rigorous methodology that involves a large number of health professionals and patients, which will strengthen the validity of the results. Although it will be difficult to control the extent to which the personal skills of the researchers might influence the success of the intervention, its format, content, and duration will be standardized in an effort to minimize this potential confounder.

## Conclusions

In conclusion, the use of spirometry as a motivational instrument for smoking cessation continues to be a controversial topic. Although the available studies seem to highlight the efficacy of this intervention, there is insufficient evidence to establish definitive conclusions about its effectiveness. The results of the present study could contribute to clarify these and other questions of practical utility for primary care professionals.

## Competing interests

The authors declare that they have no competing interests.

## Authors' contributions

FML, JBG, JLPM, and NMV form the nucleus of the team of researchers in the ESPITAP study. FML is the principal investigator and developed the original idea for the project. The study design was further developed by FML, JLPM, JBG and NMV. The following have intervened in the design and the planning of the intervention that is evaluated: FML, NMV, IPP, EAL, RSA, TBG and RPL. JLP developed the statistical methods. All authors have read and corrected draft versions, and approved the final manuscript.

## Pre-publication history

The pre-publication history for this paper can be accessed here:

http://www.biomedcentral.com/1471-2458/11/859/prepub
